# A Case of Thymic Asthma

**Published:** 1842-06

**Authors:** Benjamin Dennis

**Affiliations:** Cincinnati, Ohio


ART. II.—*A Case of Thymic Asthma.* By BENJAMIN DENNIS, M. D., of Cincinnati, Ohio.

As there are many highly respectable physicians who deny the existence of thymic asthma, the following case, which was supposed to be one of that kind, and was treated as such, may possess some interest.

In the latter part of December, 1839, I was called to see a child aged 15 months, son of Mr. C——, of Walnut street, who, according to the unsatisfactory history of the case, as gleaned from its mother, had been for the five previous months troubled with difficult respiration, continual wheezing, and occasional attacks of dyspnœa with threatened suffocation. Up to the time just mentioned, the child had been favored with uninterrupted good health; but now, in consequence of the duration of the malady, it was greatly emaciated—reduced almost to a mere skeleton, The attacks of dyspnœa occurred as often as four or five times during twenty-four hours, and each of them created the most fearful alarm concerning its termination.

Having never before witnessed a case of thymic asthma, nor read much relating to its true character, I did not suspect the presence of that affection, but was disposed to regard the case as one of simple asthma. In this error I was not alone, as five other medical gentlemen who had visited the child before I was consulted, had arrived at the same conclusion. With this view the usual remedies applicable in the cure of asthma were prescribed; but perceiving no improvement in my little patient, I suspected that the difficulty might be the consequence of elongated uvula; upon an examination, however, this proved not to be the fact. I then determined upon a consultation.

January 10th, 1840. Visited my patient in consultation with Prof. J. P. Harrison, who, after instituting a careful inquiry into the history of the family, thereby ascertaining that they were predisposed to scrofulous affections, suggested to me that the case was one of thymic asthma. We immediately proceeded to examine the gland, which was found greatly enlarged, producing a very marked projection of the superior end of the sternum; it extended itself superiorly to the thyroid gland, and laterally to about a half-inch beyond the sternal extremity of each clavicle. The following prescription was agreed upon:

℞ Iodine ℈j.
Hyd. Potassæ, ℈ij.
Aqua distillat. ℥j.
Fiat solutio.

Two drops, to be given in a tea-spoonful of sweetened water, three times a day.

The parts over the gland were directed to be rubbed three or four times a day with salt water.

This course, attended with a gradual improvement in the health of the little patient, was continued for two months, increasing the dose of the iodic solution one drop each successive week, and substituting the ointment of iodine for the salt water.

A small portion of calomel and rhubarb, a grain of each, was prescribed at occasional periods, when the state of the bowels rendered it necessary. Plenty of fresh air and plain, but nutritious diet, were also advised.

The gland continued to diminish in size, and at the expiration of three months from the time of our visit, the health of the little sufferer was apparently completely re-established.

In February last, I was informed through a friend of the family of Mr. C., who is now living in Illinois, that the child had had no return of the affection, and that his health was uniformly good.

May, 1842.

